# Supplementary figures and images for: Progressive Supranuclear Palsy in India: Insights from a Large Multicenter Clinical Cohort (Project PAIR‐PSP)
Source: Mov Disord Clin Pract. 2026 May 24:10.1002/mdc3.70672. Online ahead of print. doi: 10.1002/mdc3.70672 (PMC13339298; doi:10.1002/mdc3.70672)

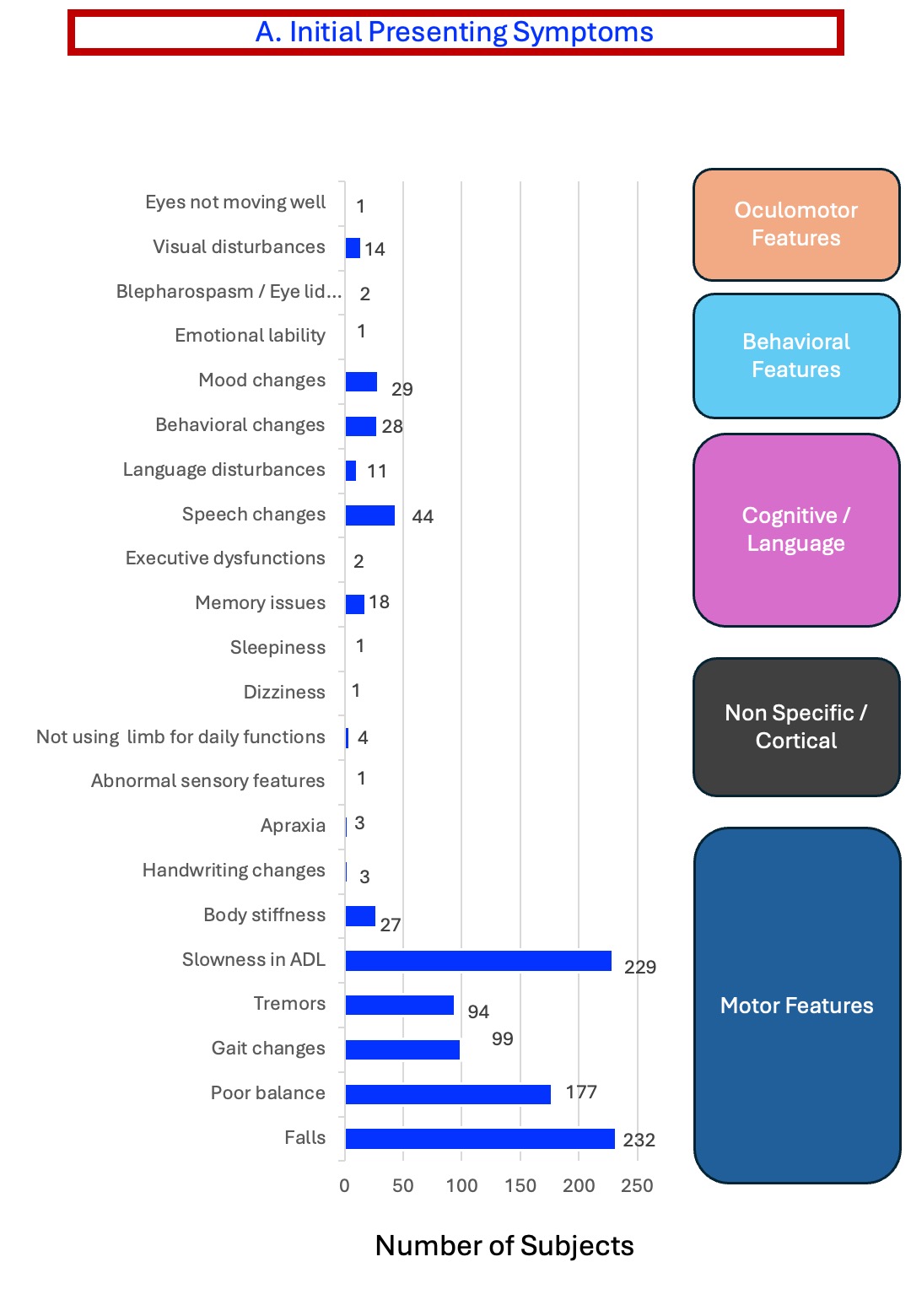

Supplement: Supplementary file 3 — Supplementary Figure A. Initial presenting symptoms at onset. [file MDC3-9999-0-s001.jpg]

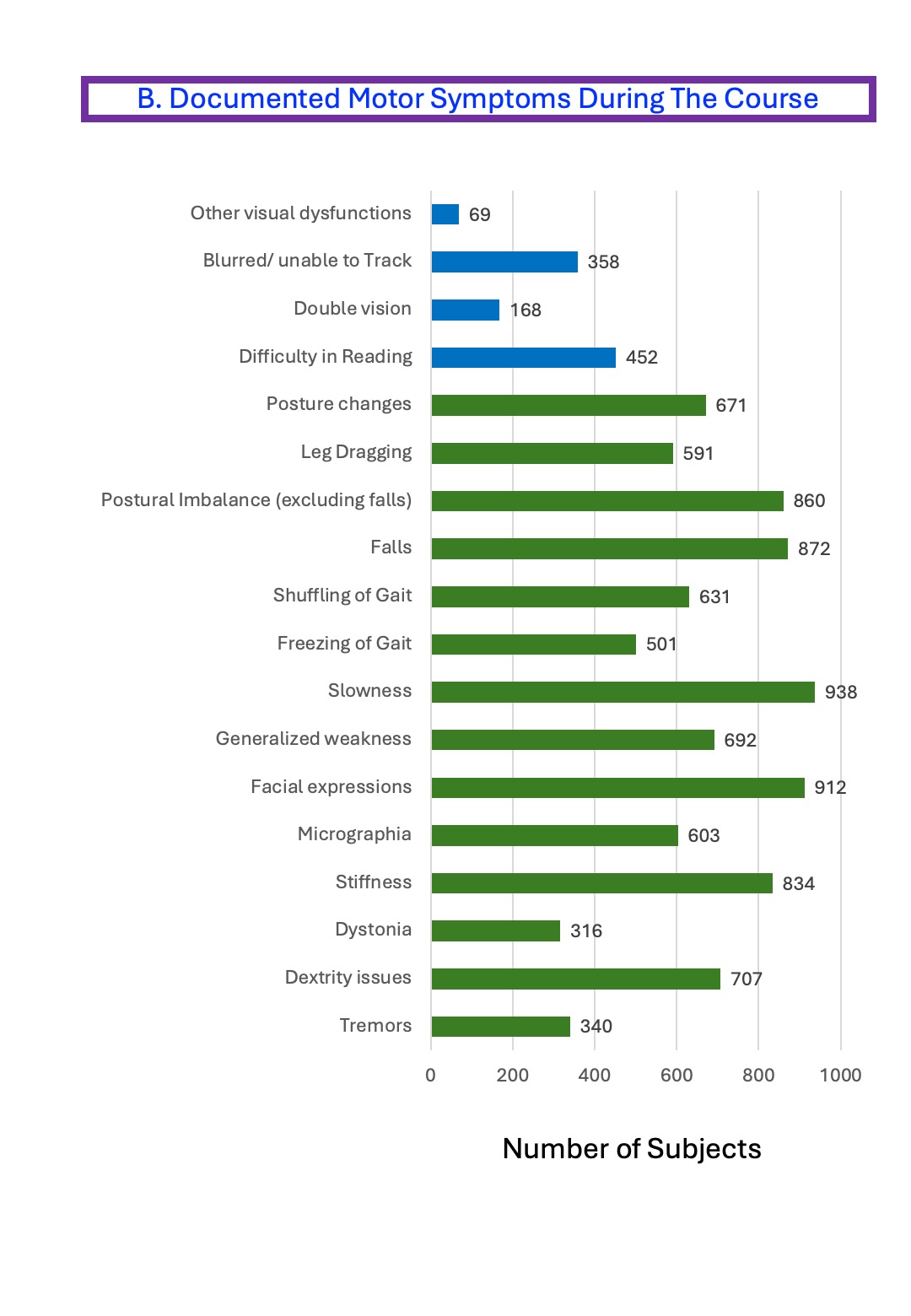

Supplement: Supplementary file 4 — Supplementary Figure B. Documented Motor and Occulomotor Symptoms till the recruitment. The list shows symptoms noted during the course of symptoms and may not indicate as presenting symptom or the symptom at the point of inclusion. The list indicates the symptoms were documented. Undocumented indicates either absence of symptoms or as data not entered. [file MDC3-9999-0-s006.jpg]

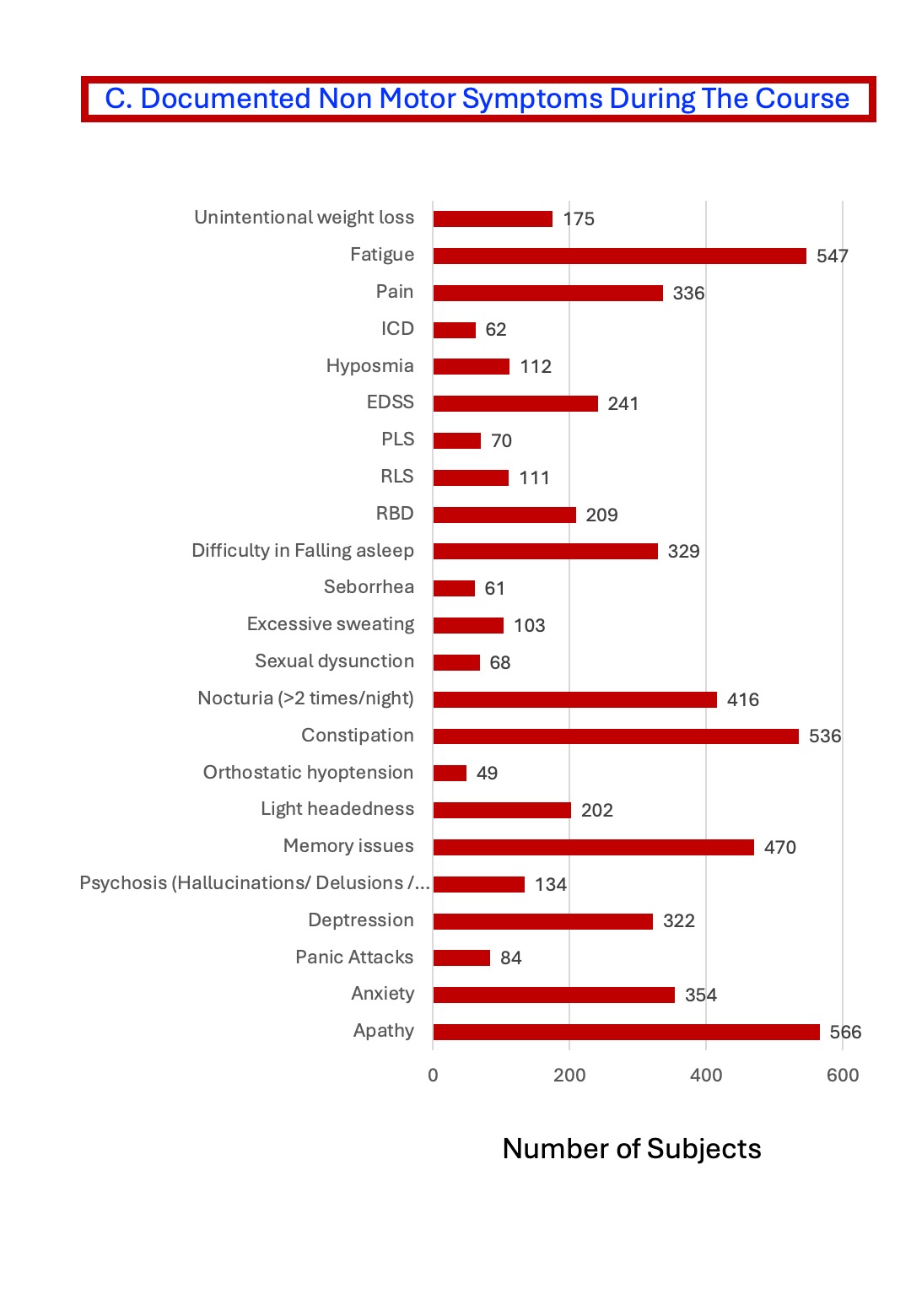

Supplement: Supplementary file 5 — Supplementary Figure C. Documented non motor symptoms during the course. The list shows symptoms noted during the course of symptoms and may not indicate as presenting symptom or the symptom at the point of inclusion. The list indicates the symptoms were documented. Undocumented indicates either absence of symptoms or as data not entered. [file MDC3-9999-0-s005.jpg]
